# RNA sequencing reveals novel LncRNA/mRNAs co-expression network associated with puerarin-mediated inhibition of cardiac hypertrophy in mice

**DOI:** 10.7717/peerj.13144

**Published:** 2022-04-05

**Authors:** Shan Ye, Weiyan Chen, Caiwen Ou, Min-Sheng Chen

**Affiliations:** 1 Department of Cardiology, Laboratory of Heart Center, Guangdong Provincial Biomedical Engineering Technology Research Center for Cardiovascular Disease, Sino-Japanese Cooperation Platform for Translational Research in Heart Failure, Zhujiang Hospital, Southern Medical University, Guangzhou, PR China; 2 Department of Geriatrics, The Second Affiliated Hospital of Guangzhou Medical University, Guangzhou, Guangdong, China; 3 Intensive Care Unit, The Second Affiliated Hospital of Guangzhou Medical University, Guangzhou, Guangdong, China; 4 Dongguan Hospital of Southern Medical University, Dongguan, Guangdong, China

**Keywords:** Long non-coding RNA, Message RNA, RNA sequencing, Cardiac hypertrophy, Puerarin

## Abstract

**Background:**

Evidence has demonstrated that puerarin is a potential medicine for the treatment of cardiac hypertrophy. However, the precise underlying molecular mechanisms of the protective effect of puerarin are still unclear. Here, we aimed to explore the regulatory mechanisms of lncRNAs/mRNAs co-expression network in a cardiac hypertrophy mouse model after puerarin treatment.

**Methods:**

A mouse model of cardiac hypertrophy was established by transverse aortic constriction (TAC). The echocardiography, tissue staining and western blot were used to examine the protective effect of puerarin. Then RNA sequencing (RNA-seq) was carried out to analyze systematically mRNAs and lncRNAs expression. The target lncRNA were confirmed using qRT-PCR. Moreover, a coding/non-coding gene co-expression network were established to find the interaction of lncRNA and mRNAs. The biological process, cellular component, molecular function and pathways of different expression mRNAs targeted by lncRNA were explored using Gene Ontology (GO) analysis and Kyoto Encyclopedia of Genes and Genomes (KEGG) pathways analysis.

**Results:**

Puerarin exhibited an obvious inhibitory effect in cardiac hypertrophy in TAC model. RNA-seq analysis was performed to investigate the lncRNAs and mRNAs expression patterns of cardiomyocytes in sham and TAC groups treated with or without puerarin. RNA-seq identified that TAC downregulated four lncRNAs, which could be revised by puerarin treatment (|log2 Fold change| > 2 and FDR < 0.05). Among them, expression alterations of lncRNA Airn (antisense of Igf2r non-protein coding RNA) was confirmed by qRT-PCR. Pearson’s correlation coefficients of co-expression levels suggested that there was an interactive relationship between Airn and 2,387 mRNAs (*r* > 0.95 or *r* < −0.95). Those co-expressed mRNAs were enriched in some important biological processes such as translational initiation, cell proliferation, insulin-like growth factor binding and poly(A) RNA binding. KEGG analyses suggested that those Airn-interacted mRNAs were enriched in endocytosis, signaling pathways regulating pluripotency of stem cells and the Jak-STAT pathway.

**Conclusion:**

Puerarin may exert beneficial effects on cardiac hypertrophy through regulating the lncRNAs/mRNAs co-expression network.

## Introduction

Left ventricular hypertrophy refers to the enlargement and thickening of the left ventricle walls. Developing left ventricular hypertrophy puts people at higher risk of myocardial infarction, heart failure and cardiac arrest (Mongiovi et al., 2010; Tang et al., 2020). It is demonstrated that cardiomyocyte hypertrophy is the main pathological change of left ventricular hypertrophy (Hutchings et al., 2018). However, there is no effective treatment for myocardial hypertrophy so far (Rodrigues, Leite-Moreira & Falcao-Pires, 2016). Therefore, it is urgent to find an effective treatment for cardiomyocyte hypertrophy. Puerarin (PUE) is an active component isolated from the Traditional Chinese Medicine Gegen (Zhang, 2019). Recently, it was found that puerarin could significantly inhibit cardiomyocyte hypertrophy. It was reported that puerarin prevented cardiac hypertrophy through regulating the AMPK, mTOR and Nrf2 pathway, suggesting that puerarin may be potential drug candidate for myocardial hypertrophy (Cai et al., 2018; Liu et al., 2015). However, the effect of puerarin on cardiomyocyte hypertrophy and its precise underlying molecular mechanisms need further study before its clinical usage for myocardial hypertrophy.

Long non-coding RNA (lncRNA) is a kind of non-coding RNA with a length of more than 200 nucleotides (Statello et al., 2021b). Although lncRNAs do not code for proteins, they exert regulatory functions at various levels of gene expression, including chromatin modification, transcription, and post-transcription (Statello et al., 2021a, 2021b; Yang et al., 2020). Recently, the role of lncRNA in cardiovascular disease has attracted more and more attention. Many previous studies have found that lncRNA is abundant in the cardiovascular system, which is involved in the pathophysiological processes of heart development, myocardial remodeling, myocardial hypertrophy and cardiomyocyte apoptosis, *etc*. (Diao & Zhang, 2021; Gomes et al., 2020; Zhang, Yang & Qiu, 2021). Although current studies have demonstrated that lncRNAs play an important part in the pathophysiological process of cardiac disease, whether lncRNAs are associated with the protective role of puerarin in myocardial hypertrophy remains unknown. In the present study, RNA sequencing (RNA-seq) was performed to understand systematically the function of lncRNAs in the pharmacological action of puerarin in myocardial hypertrophy.

## Materials and Methods

### Study design and transverse aortic constriction (TAC)-induced myocardial hypertrophy mouse model

Male C57BL/6 mice (aged 5 weeks old) used in this study were purchased from the Guangdong Medical Laboratory Animal Center (Guangzhou, China). The mice were fed a standard laboratory diet and housed under standard conditions. Two weeks later, a total of 15 mice were randomly assigned using computer-generated random number tables to sham group, TAC group and TAC+PUE group. The sham group mice received intraperitoneal (i.p.) injection of the saline and the surgical procedures without the constriction. TAC group mice received i.p. injection of the saline and the TAC procedures to induce cardiac hypertrophy. The TAC+PUE group mice received a daily i.p. injection of puerarin (Sigma Aldrich, St. Louis, MO, USA) 3 days before TAC and continued for 21 days after TAC. The doses administered for puerarin (100 mg/kg per day) were chosen based on previous studies (Chen et al., 2014).

TAC is widely used as a disease model of chronic ventricular hypertrophy (Bosch et al., 2020). Thereby, TAC was used to establish myocardial hypertrophy in mice as described in a previous study (Meng et al., 2018). Briefly, following anesthetization with isoflurane (Henry Schein, Melville, NY, USA), the aortic arch was exposed after a midline incision in the anterior neck. The transverse aortic arch between the left common carotid artery and the brachiocephalic artery was chosen as the site of constriction. The aortic arch was constricted by tying a 6.0 nylon suture ligature against a 26-gauge needle. After rapid removal of the needle, an incomplete constriction was formed. The successful constriction of TAC was verified using trans-thoracic echocardiography. All procedures were carried out according to the Guide for the Care and Use of Laboratory Animals (National Research Council (U.S.) et al., 2011). All animal experiments were approved by the Institutional Animal Care and Use Committee of the Second Affiliated Hospital of Guangzhou Medical University, Guangzhou, China (No. B2019-064).

### Organ weight

Body weight (BW) and tibia length (TL) of each mouse were measured 21 days after the TAC procedure. The mice were sacrificed using cervical dislocation under aesthesia, and hearts were arrested in diastole with injection of potassium chloride. And then, the hearts were gained and heart weight (HW) were measured. Heart weight to body weight ratio (HW/BW) and heart weight to tibia length ratios (HW/TL) were counted.

### Echocardiography

After anesthetization with isoflurane, successful ligation was confirmed by color Doppler and pulsed-wave Doppler scanning. Left ventricular posterior wall dimension (LVPWd) and interventricular end-diastolic septum thickness (IVSd) were measured by two-dimensional transthoracic echocardiography. The transthoracic echocardiography was performed by an experienced technician who was blinded to the study groups using an IE33 echocardiographic system (Philips Medical Systems, Leiden, the Netherlands).

### Hematoxylin-eosin (HE) staining

After fixation with 10% formalin, the heart tissues were dehydrated through a serial alcohol gradient and embedded in paraffin wax blocks. And then, heart sections were stained with HE solution (Beyotime, China). The sections were examined under a light microscope (Nikon Technology Co., Ltd., Japan).

### Western blot analysis

Western blot analysis was performed as described previously (Lin et al., 2018, 2021). Briefly, the whole protein of the heart was extracted using radio-immunoprecipitation assay (RIPA) lysis buffer (Kaiji Company, Shen Zhen, China) with protease and phosphatase inhibitors (Kaiji Company, Shen Zhen, China). Protein samples were separated by sodium dodecylsulphate polyacrylamide gel electrophoresis (SDS-PAGE) and transferred to polyvinylidene difluoride (PVDF) membranes. The membranes were then incubated at 4 °C overnight with the following primary antibodies: β-MHC (Bioworld Technology Inc., Louis Park, MN; USA; BS70815, 1:1,000) and GAPDH (Cell Signaling Technology Inc., Danvers, MA, USA; # 5174, 1:1,000). After being washed three times with PBST, the membranes were incubated with the secondary antibodies for 1 h at room temperature. Then, the signals were detected using the Imaging System (GE, Amersham Imager 600, GE, Piscataway, NJ, USA). The relative expression level of proteins was analyzed using Image-Pro Plus 6.0 (Media Cybernetics Inc., Bethesda, MD, USA).

### RNA extraction

Three mice in each group were randomly selected to harvest the heart for the RNA sequence analysis. The mice were sacrificed at 21 days after TAC and hearts were harvested. Total RNA was isolated from heart tissues using Trizol reagent (TaKaRa, Tokyo, Japan). The concentration of the RNA samples was evaluated using a NanoDrop ND-1000 instrument (Thermo Fisher Scientific, Waltham, MA, USA). The integrity of the RNA was assessed by electrophoresis on an agarose gel.

### RNA sequencing analysis

Library preparation and Illumina sequencing analysis were performed as previous study (Li et al., 2017). Briefly, the RNA of heart tissue was used for library construction by KAPA Stranded RNA-seq library Prep Kit (Illumina, NEB, San Diego, CA, USA). Then, the library was pair-end sequenced by Illumina NovaSeq 6000 Sequencing system (Illumina, NEB, San Diego, CA, USA) according to the manufacturer’s protocol. The whole-transcriptome sequencing experiment was completed by Kangcheng Biotechnology Co., Ltd. (Shanghai, China). The per base quality scores indicating the sequences data is of high quality. After trimming the adaptor sequences, clean sequences reads were obtained for subsequently analysis. Then the clean reads were mapped to a reference genome using Hisat2 software. The transcripts were assembled and quantitated by StringTie. The expression level of transcripts was measured by transcripts per kilobase million (TPM) values using the R package of Ballgown (Meng et al., 2021; Zhao et al., 2021b). The unknown transcripts were further screened with Coding Potential Assessing Tool (CPAT) to distinguish the protein-coding genes from the non-coding genes. The unknown non-coding transcripts were then used to screen for novel lncRNAs. The unknown non-coding transcripts with lengths more than 200 nt and more than two exons were selected as novel lncRNA candidates (Zhao et al., 2021a). Raw sequence files have been deposited at NCBI’s Gene Expression Omnibus (Accession code: GSE176244). Differentially expressed lncRNAs with statistical significance among the three groups were observed through volcano plot filtering. Hierarchical clustering was conducted to demonstrate the distinguishable lncRNAs expression pattern among the groups. The volcano plot filtering and hierarchical clustering analysis was conducted from the TPM values using the R package of ggthemes and pheatmap accordingly.

### Quantitative real-time polymerase chain reaction (qRT-PCR) analysis

The lncRNA-seq results were further validated by qRT-PCR analysis as previous study (Lin et al., 2020). Briefly, total RNA isolated from heart tissues was reverse transcribed to synthesize cDNA using SuperScriptTM III Reverse Transcriptase kit (Invitrogen: 18080-044). Then, the qRT-PCR was performed by the QuantStudio5 Real-time PCR System (Applied Biosystems, Waltham, MA, USA) with the 2 × PCR Master Mix (Arraystar: AS-MR-006-5). Airn (antisense of Igf2r non-protein coding RNA) expression was determined using the 5′-CTAAGCCACTGCCGTCCATA-3′ forward primer and 5′-TAGGCGTTCACCCTTGGTCT-3′ reverse primer. Relative expression levels of Airn were normalized with GAPDH (Forward primer: 5′-CACTGAGCAAGAGAGGCCCTAT-3′, Reverse primer: 5′-GCAGCGAACTTTATTGATGGTATT-3′). The results were calculated using the 2^–ΔΔCt^ method. All groups had three independent samples and all of the samples were conducted in triplicate. Cq values obtained from the RT-qPCR testing were considered acceptable for technical triplicates if they fell within mean Cq ± 2 standard deviations (SD).

### Construction of the lncRNA-mRNA co-expression network

The coding/non-coding gene co-expression (CNC) network profile was constructed according to validated altered lncRNA and its related mRNAs. The CNC was established through the weighted gene co-expression network analysis (WGCNA) using the R package WGCNA (v1.69) (Langfelder & Horvath, 2008). The co-expression network between mRNA and lncRNA was constructed utilizing Cytoscape version 2.8.1 software (The Cytoscape Consortium, San Diego, CA, USA) based on the Pearson correlation analysis of lncRNA and mRNA (*r* > 0.95 or *r* < −0.95).

### Gene function analysis

To explore the function of the selected genes in the co-expression network, Gene Ontology (GO) and Kyoto Encyclopedia of Genes and Genomes (KEGG) pathway enrichment analysis for the targets genes were implemented using Database for Annotation, Visualization and Integrated Discovery (DAVID) (Lin et al., 2016).

### Statistical analysis

All results were presented as the mean ± SD. Statistical analysis was performed using GraphPad Prism 7.0 software (San Diego, CA, USA). One-way ANOVA followed by Tukey’s *post hoc* analysis was used to test the differences among groups. A *P* value less than 0.05 was considered statistically significant.

## Results

### Puerarin inhibited TAC induced-cardiac hypertrophy in mics

**Figure 1:**
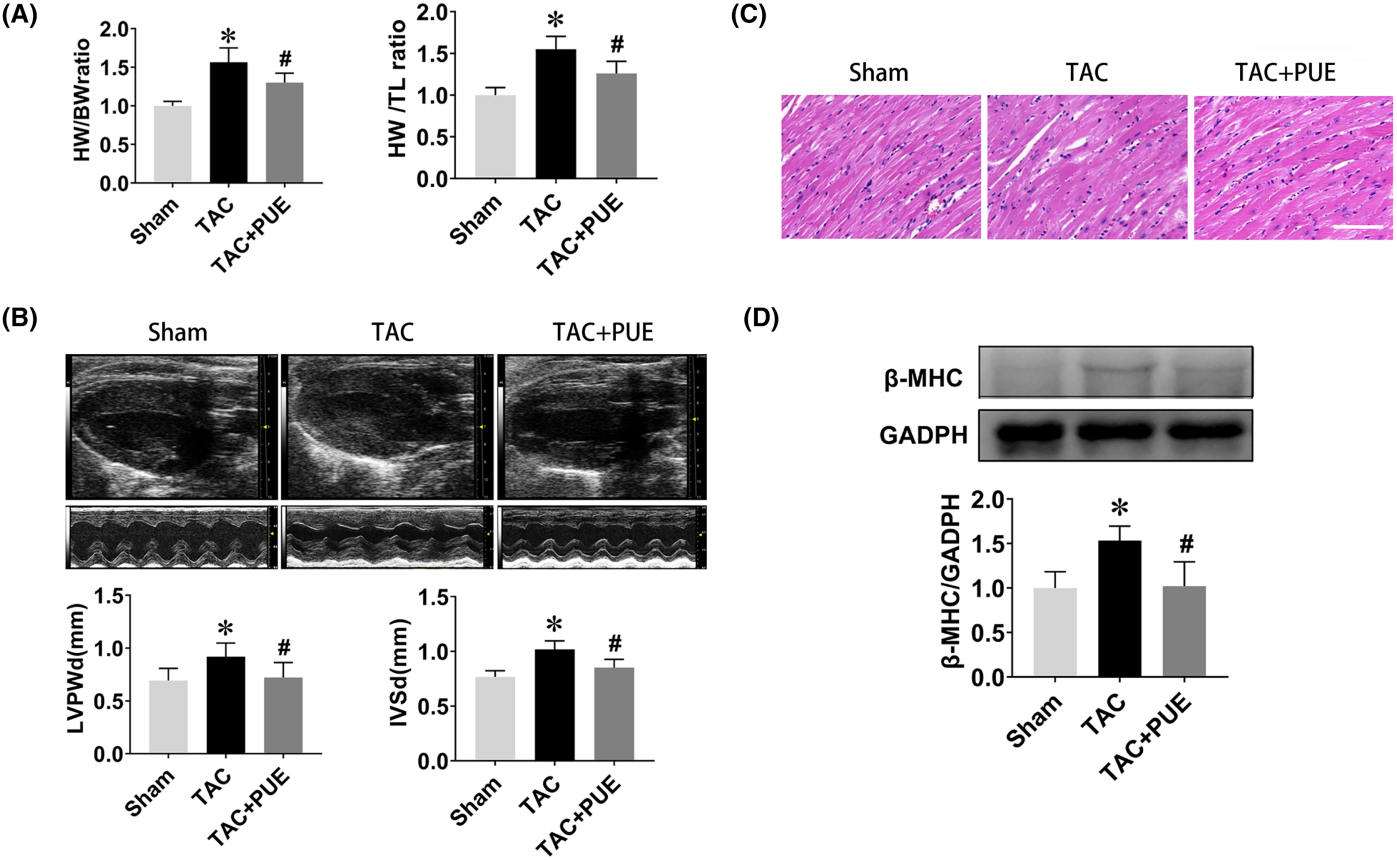
Puerarin decreased the cardiac hypertrophy induced by TAC in mice. (A) Heart weight/body weight (HW/BW) ratio and heart weight/tibial length (HW/TL) ratio from sham and TAC groups treated with or without puerarin (*n* = 5 each group). (B) Left ventricular posterior wall diameter in diastole (LVPWd) and interventricular septum diameter in diastole (IVSd) measured by echocardiography (*n* = 5 each group). (C) Representative images of HE staining (Scale bar = 100 μm). (D) Protein levels of β-MHC was detected by western blotting (*n* = 3 each group). *P < 0.05 *vs* the sham group. ^#^*P* < 0.05 *vs* the TAC group.

The ratio of heart weight to body weight or tibia length are useful indexes to quantify cardiac hypertrophy (Yin et al., 1982). As shown in Fig. 1A, the TAC group had significant high heart weight/body weight (HW/BW) ratio and heart weight/tibial length (HW/TL) ratio compared with sham group. Puerarin treatment resulted in a significant reduction of HW/BW and HW/TL ratio compared with TAC group. Echocardiography demonstrated that TAC resulted in the increase of LVPWd and IVSd, which were significantly reversed by puerarin (Fig. 1B). Besides, HE staining analysis of cardiac sections showed that the increased myocyte area in TAC mice was minimized by treatment with puerarin (Fig. 1C). Beta-myosin heavy chain (β-MHC) is a major component of cardiac myosin. The protein expression of β-MHC has historically been used as a marker of cardiac hypertrophy (Ding et al., 2019). Protein levels of β-MHC were detected by western blotting in this study. As expected, the protein expression of β-MHC increased dramatically in TAC mouse ventricular myocytes. Moreover, puerarin significantly reduced the β-MHC expression compared with TAC group (Fig. 1D).

### lncRNA expression profiles and validation

**Figure 2:**
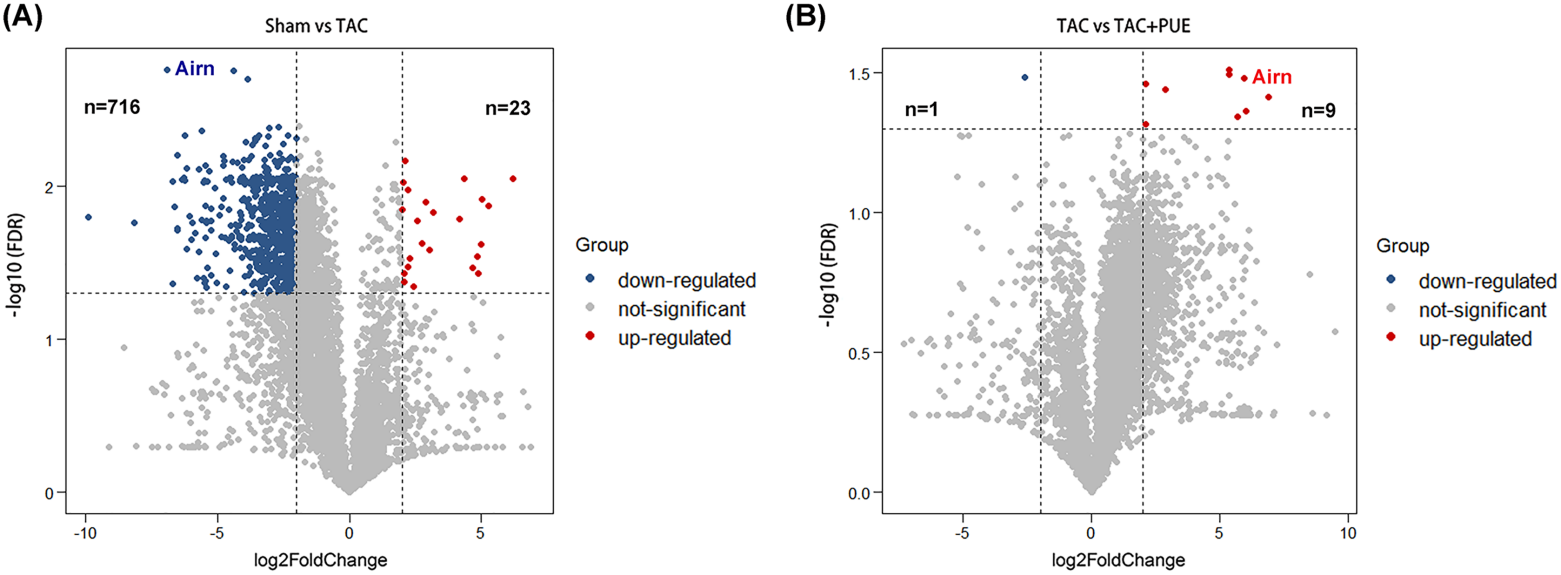
Analysis of lncRNAs expression using RNA-seq data. (A) Volcano plot displaying differentially expressed lncRNAs between the Sham and TAC groups. (B) Volcano plot displaying differentially expressed lncRNAs between the TAC and TAC+PUE groups. Red spots denote up-regulated lncRNAs and blue spots represent down-regulated lncRNAs (|log2 Fold change| > 2 and FDR < 0.05). Grey spots represent lncRNAs with no significant differences.

**Figure 3:**
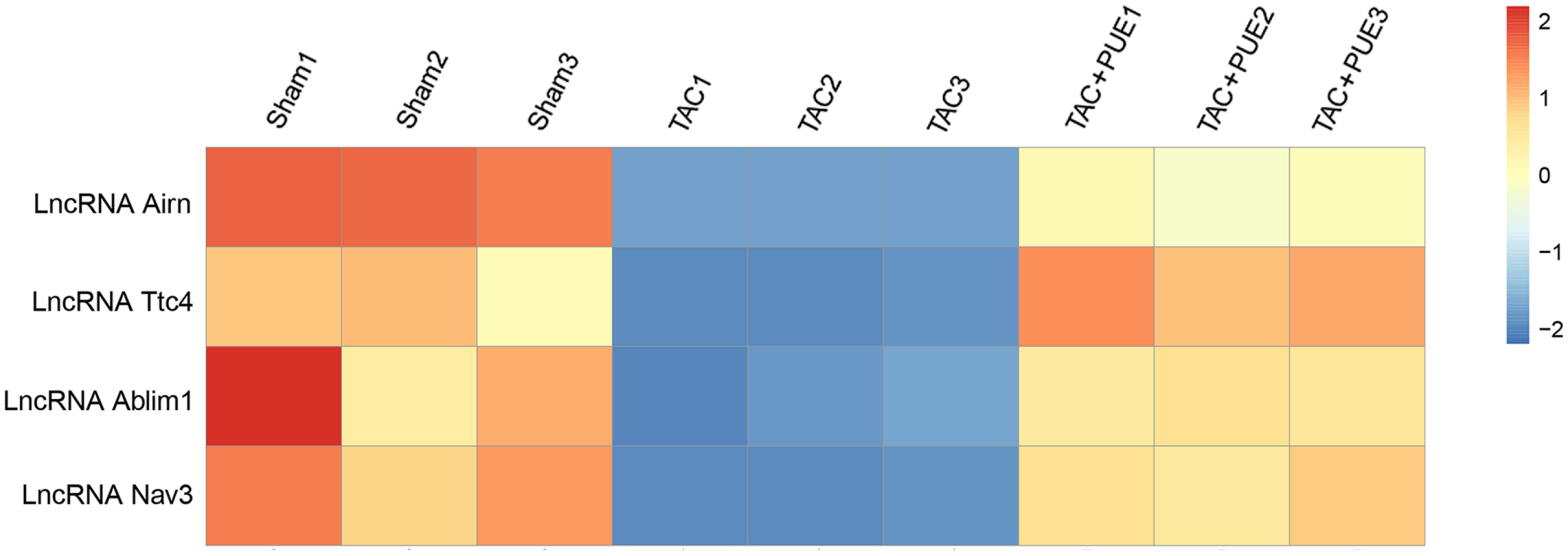
Altered lncRNAs expressions among the groups. There were four lncRNAs down-regulated in TAC group, which was reversed by puerarin treatment. Up-regulated lncRNAs are shown in red, and down-regulated lncRNAs are shown in blue.

RNA-seq was used to assess the expression levels of lncRNAs in heart samples of the sham, TAC and TAC+PUE groups. Overall, 32,105 lncRNAs were identified by RNA-seq among the groups. It was found that 23 lncRNAs were up-regulated and 716 were down-regulated in the TAC group compared with sham group (|log2 Fold change| > 2 and FDR < 0.05) through a volcano map (Fig. 2A). In addition, nine lncRNAs were up-regulated and one were down-regulated in TAC+PUE group compared with TAC group (|log2 Fold change| > 2 and FDR < 0.05) (Fig. 2B). Then, heat map showed that four lncRNAs downregulated in the TAC group were up-regulated by puerarin treatment (Fig. 3).

**Figure 4:**
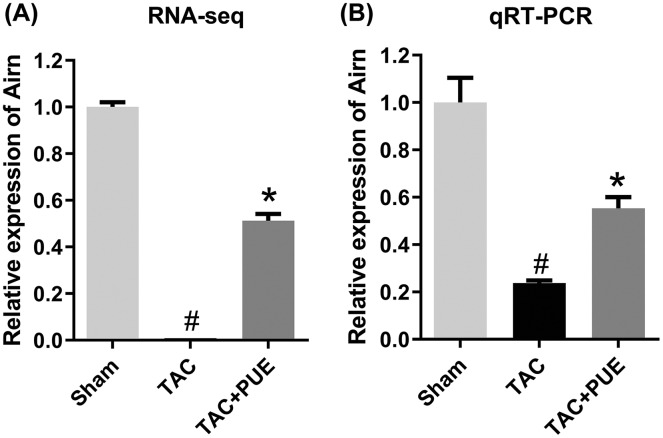
The quantitative real-time PCR (qRT-PCR) validation of differentiation expressed lncRNA in RNA-seq (*n* = 3 each group). (A) The RNA-seq results among the groups in Airn. (B) qRT-PCR results among the groups in Airn. ^#^*P* < 0.05 *vs* the sham group. *P < 0.05 *vs* the TAC group.

To validate the RNA-seq results, the lncRNA with most significant FDR value from the differentially expressed lncRNA profile was selected for the qRT-PCR analysis. The qRT-PCR results showed that there was significant difference among the groups in lncRNA Airn (*P* < 0.05) (Fig. 4).

### lncRNA-mRNA network analysis

**Figure 5:**
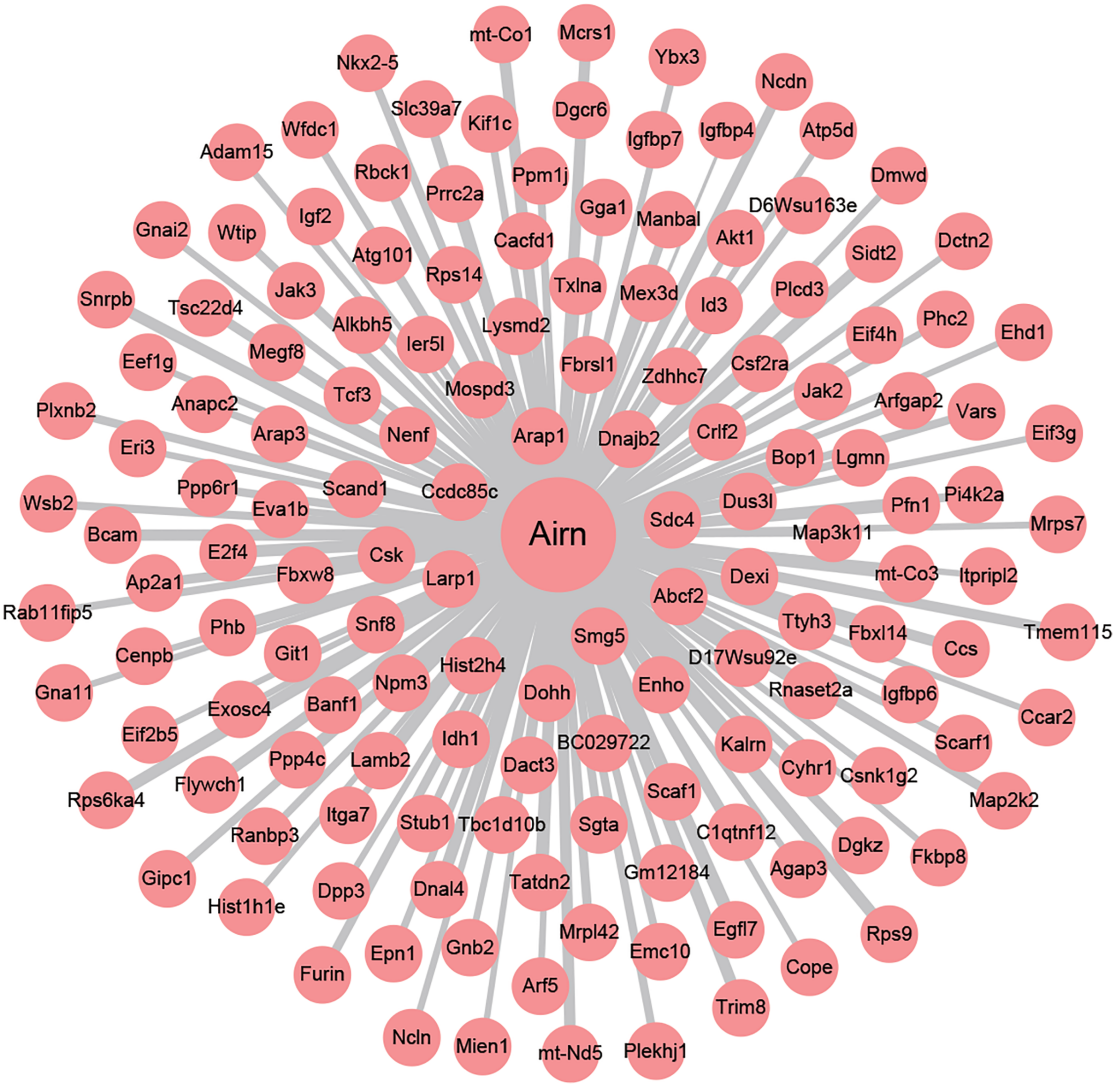
The co-expression network profiles of Airn and mRNAs. Line thickness represents the strength of the association between Airn and mRANs.

To investigate the underlying regulating mechanisms of Airn in cardiac hypertrophy and the therapeutic target of puerarin, we analyzed the co-expression of Airn and protein-coding genes by WGCNA. Pearson’s correlation coefficients of co-expression levels suggested that there was an interactive relationship between Airn and 2,387 mRNAs (*r* > 0.95 or *r* < −0.95). Those co-expressed genes including some important coding genes (Srf, Akt, Igf2, *etc*.) which have been reported to be involved in the pathogenesis of cardiac hypertrophy. We used Airn and the top 150 mRNAs co-expression with Airn to construct a lncRNA-mRNA visualization network using Cytoscape (Fig. 5).

**Figure 6:**
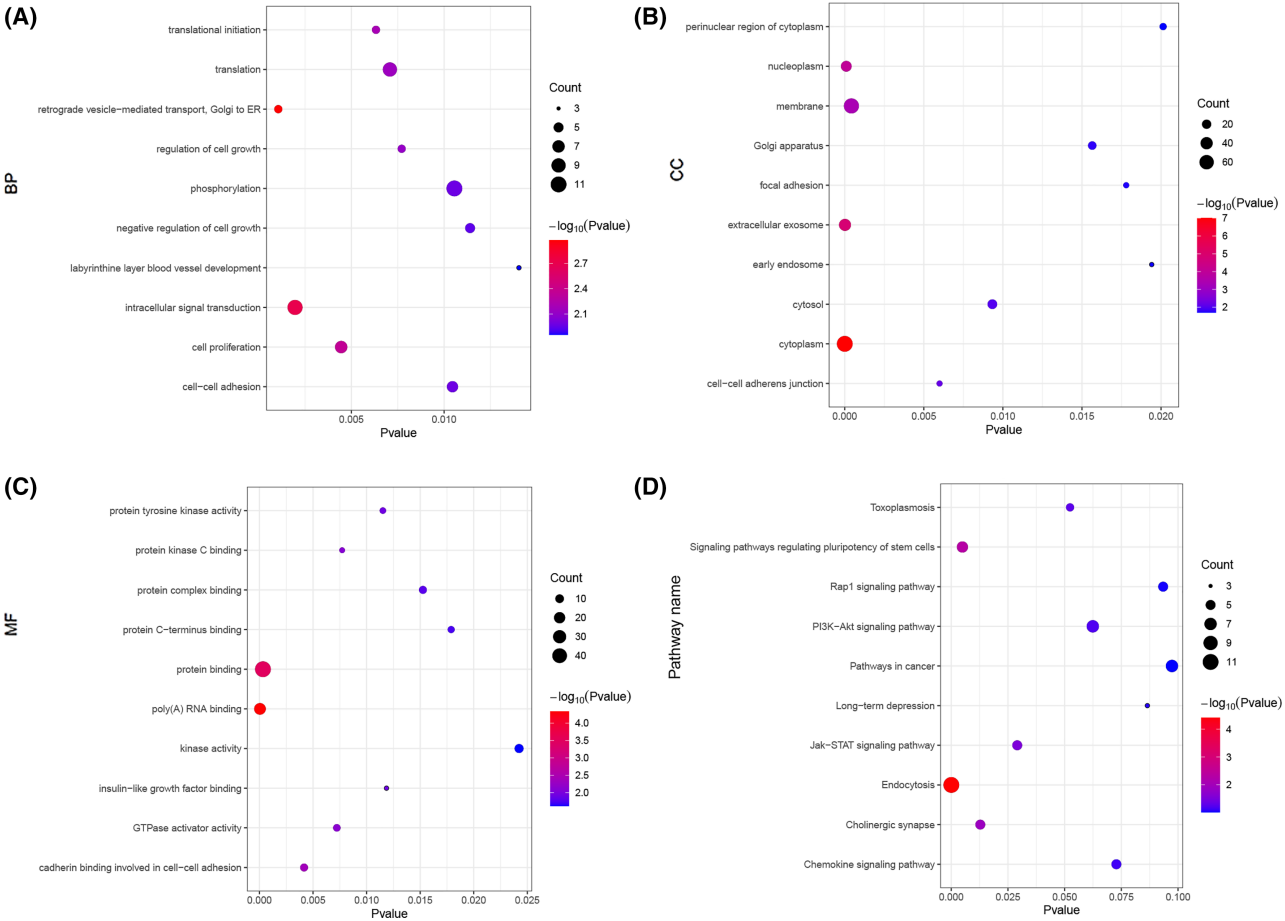
The GO and KEGG analyses of target mRNAs. (A) The top 10 significant biological process (BP) of GO analyses. (B) The top 10 significant cellular component (CC) of GO analyses. (C) The top 10 significant molecular function (MF) of GO analyses. (D) The top 10 significant enriched pathways by KEGG pathway analyses. The node size shows the number of genes, and the color scale represents the –log10(*P* value).

Next, we carried out a functional enrichment analysis of the top 150 target genes using over-representation analysis. GO analysis found that these target mRNAs were enriched in some important biological processes and molecular function such as translational initiation, cell proliferation, insulin-like growth factor binding and poly(A) RNA binding (Figs. 6A–6C). KEGG analyses suggested that those Airn-interacted mRNAs were enriched in endocytosis, signaling pathways regulating pluripotency of stem cells and Jak-STAT pathway (*P* < 0.05) (Fig. 6D).

## Discussion

In the present study, we comprehensively investigated the role of lncRNAs in the cardioprotection of puerarin through RNA-seq. RNA-seq revealed that TAC downregulated four lncRNAs, which were reversed by puerarin treatment. Among them, Airn were verified by qRT-PCR. This data suggests that lncRNAs might be involved in the cardioprotection of puerarin.

It was found that lncRNAs were significantly altered in the mouse model of cardiac hypertrophy, suggesting that lncRNAs might play an important role in the pathogenesis of cardiac hypertrophy (Wang et al., 2020). Previous studies demonstrated critical roles of particular lncRNAs such as Mhrt, Chaer and MEG3 in TAC mice. Those lncRNAs are proved to be candidates in the pathologic progression of hypertrophy cardiomyopathy (Liu, Zhang & Li, 2020; Xu et al., 2020). Our RNA-sequence results also found those dysregulated lncRNAs in cardiac tissue, and the change trend of those lncRNAs between the Sham and TAC groups was consistent with previous reports (Han et al., 2014; He et al., 2021; Liu, Zhang & Li, 2020; Piccoli et al., 2017). However, those three dysregulated lncRNAs could not be reversed by puerarin according to our sequencing results.

We found that lncRNA Airn, lncRNA Ttc4, lncRNA Ablim1 and lncRNA Nav3 were novel lncRNAs associated with cardiac hypertrophy. Moreover, we found that those dysregulated lncRNAs could be reversed by puerarin. Thus, those dysregulated lncRNAs might be promising therapeutic targets to suppress cardiac hypertrophy (Wang et al., 2020). Importantly, those four lncRNAs might be involved in puerarin-mediated inhibition of cardiac hypertrophy in mice.

Airn is a paternally expressed imprinted gene (Santoro et al., 2013). It located in antisense orientation to the maternally expressed Igf2r (insulin-like growth factor 2 receptor) gene (Latos et al., 2012). Although it is known that Airn and Igf2r are mutually imprinted, up until now, the functions of Airn still need to be explored, especially in the cardiovascular system. A recent study reported that knockdown of Airn leaded to cardiomyocytes injury by reduced translation of Igf2bp2 (insulin-like growth factor 2 mRNA-binding protein 2) protein (Hosen et al., 2018). In this study, we found that the decreased expression of Airn is associated with cardiac hypertrophy in mice. In addition, we used the co-expression of Airn and protein-coding genes from the RNA-seq to investigate the underlying regulating mechanisms of Airn in cardiac hypertrophy and the therapeutic target of puerarin. Through WGCNA and Gene Ontology analysis, it was found that Airn was co-expressed with the insulin-like growth factor signalling pathway-related genes (Igf2, Igfbp4, Igfbp6, *etc*.). The result demonstrated that Airn might affect insulin-like growth factor signalling pathway-related genes and contribute to the cardiac hypertrophy in mice, which is consist with previous study (Diaz Del Moral et al., 2021; Hosen et al., 2018). The insulin-like growth factor pathway-related genes have been reported to play an important role in the occurrence and development of myocardial hypertrophy (Lu et al., 2021; Nomura et al., 2018; Zhang et al., 2021). The role of the Airn/insulin-like growth factor signalling pathway in cardiac hypertrophy, and whether this pathway is a drug target for puerarin in the treatment of cardiac hypertrophy needs to be further studied.

The limitation of this study is the lack of functional assessment of the identified lncRNA and mRNAs. Further mechanisms study will provide us a comprehensive understanding of puerarin pharmacological activities, which will be helpful for the development of clinical patient treatment and clinical practice guidelines.

In conclusion, our study used deep RNA-seq analysis to reveal that lncRNAs might be involved in the cardioprotection of puerarin. This study provides novel insights into our understanding of the pathogenesis of cardiac hypertrophy and proves the therapeutic role of puerarin in preventing or reversing cardiac hypertrophy.

## Supplemental Information

10.7717/peerj.13144/supp-1 Supplemental Information 1 Raw Western Blot images: GADPH.

10.7717/peerj.13144/supp-2 Supplemental Information 2 Raw Western Blot images: β-MHC.

10.7717/peerj.13144/supp-3 Supplemental Information 3 All Differentially Expressed LncRNA.

## Additional Information and Declarations

### Data Availability

The following information was supplied regarding data availability: The data is available at the NCBI Gene Expression Omnibus: GSE176244.
