# Supplementary figures and images for: RNA sequencing reveals novel LncRNA/mRNAs co-expression network associated with puerarin-mediated inhibition of cardiac hypertrophy in mice
Source: PeerJ. 2022 Apr 5;10:e13144. doi: 10.7717/peerj.13144 (PMC8992661; doi:10.7717/peerj.13144)

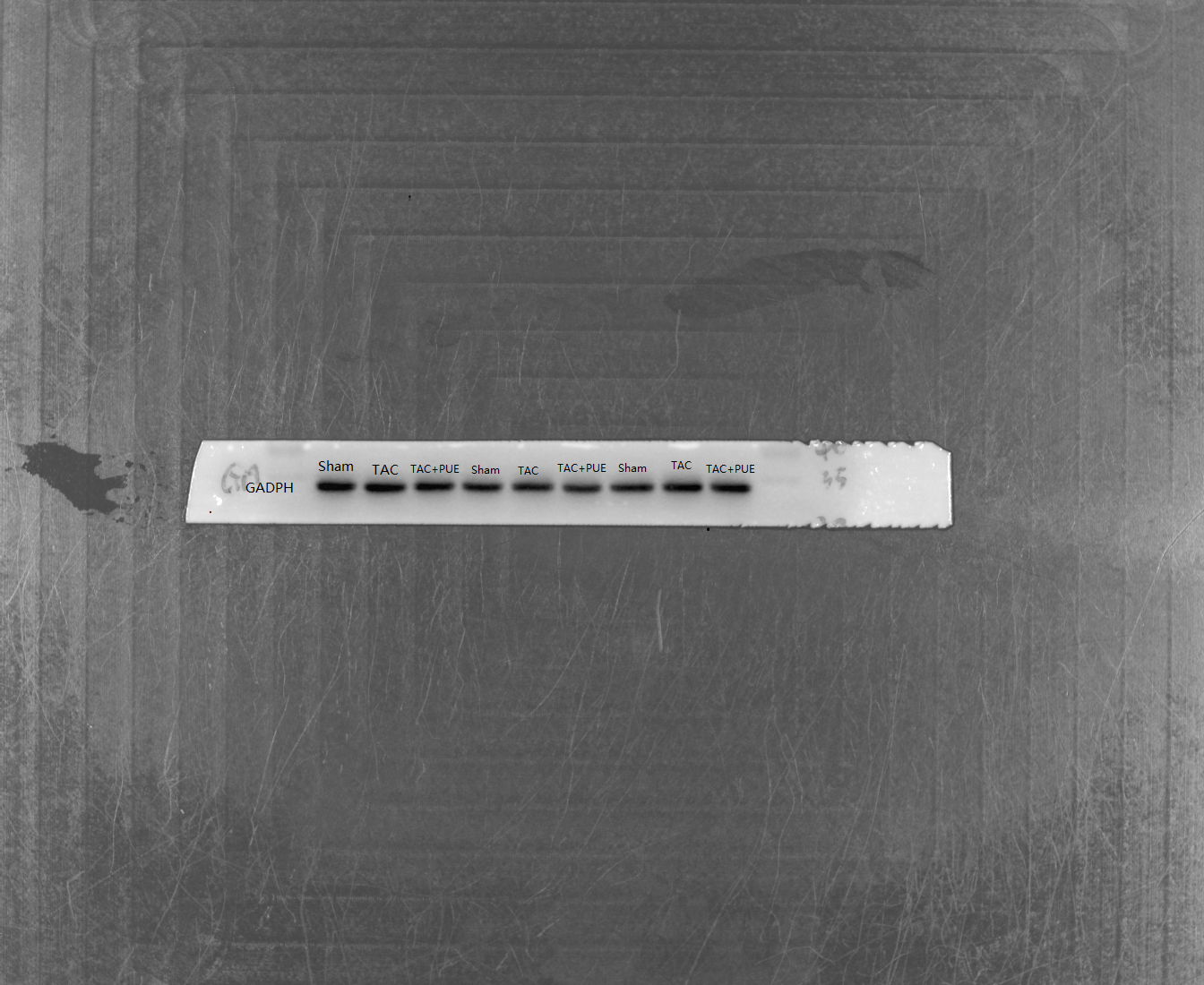

Supplement: Supplemental Information 1 [file peerj-10-13144-s001.tif]

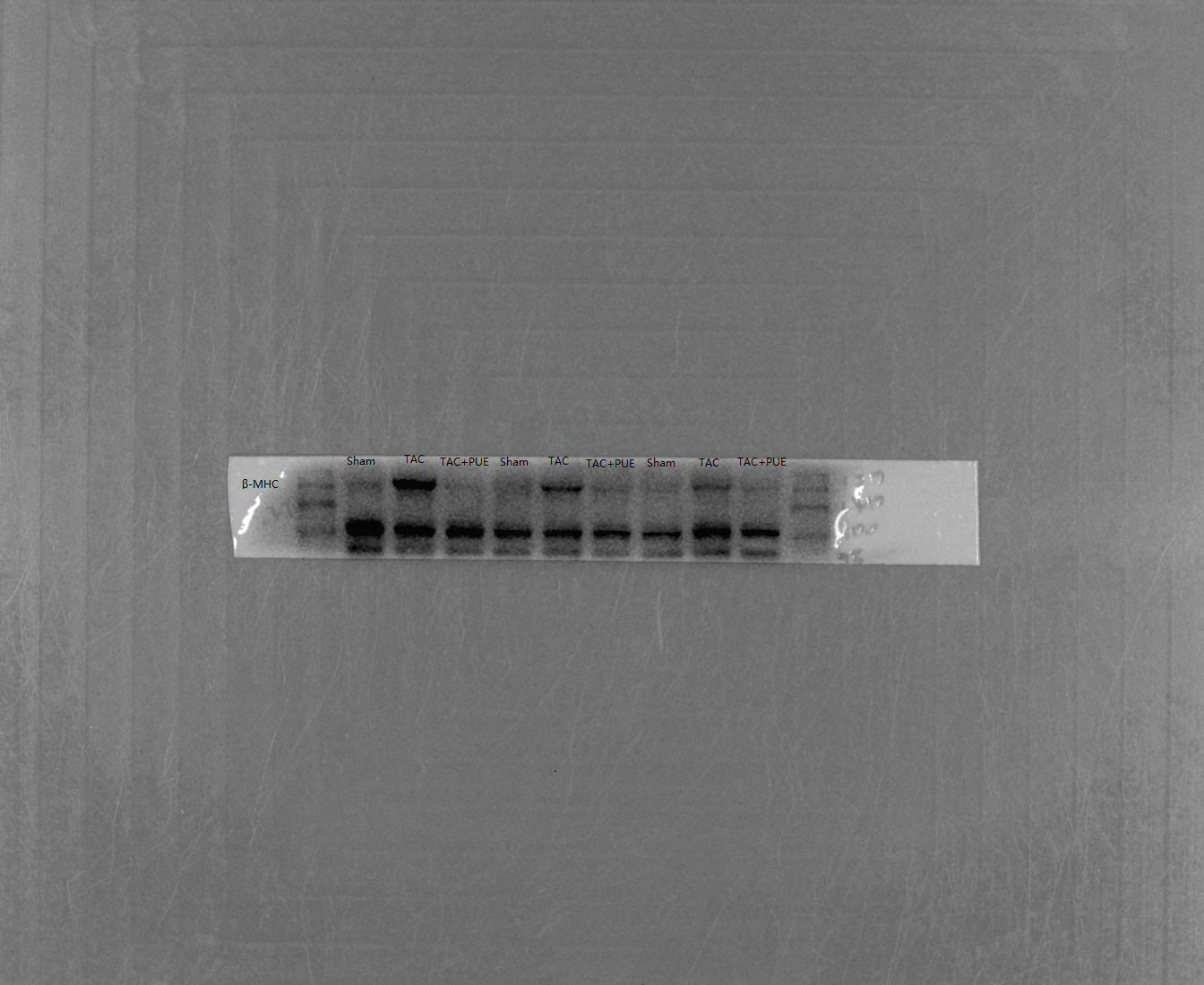

Supplement: Supplemental Information 2 [file peerj-10-13144-s002.tif]
